# Nucleic Acids on the Surface and Lumen of Tumor-Derived Small Extracellular Vesicles as Potential Cancer Biomarkers

**DOI:** 10.3390/cells15060512

**Published:** 2026-03-13

**Authors:** Alicja Gluszko, Daria Kania, Chang-Sook Hong, Monika Pietrowska, James F. Conway, Theresa L. Whiteside

**Affiliations:** 1 Department of Pathology and UPMC Hillman Cancer Center, University of Pittsburgh School of Medicine, Pittsburgh, PA 15213, USA; gluszkoa@upmc.edu (A.G.); hongcs@upmc.edu (C.-S.H.); 2 Department of Biochemistry, Medical University of Warsaw, 02-097 Warsaw, Poland; monika.pietrowska@gliwice.nio.gov.pl; 3 Maria Sklodowska-Curie National Research Institute of Oncology, 44-102 Gliwice, Poland; daria.kania@gliwice.nio.gov.pl; 4 Department of Structural Biology, University of Pittsburgh School of Medicine, Pittsburgh, PA 15261, USA; jxc100@pitt.edu

**Keywords:** tumor-derived extracellular vesicles (TEX), vesicle corona, surface DNA, luminal ds-DNA, mutated genomic DNA

## Abstract

**Background**: Tumor-derived small extracellular vesicles (sEV), which we call TEX, carry a cargo of molecules that resembles the producer tumor cells. Circulating freely in body fluids, TEX potentially serve as a liquid tumor biopsy. TEX horizontally transfer their cargo to various recipient cells, imparting to them pro-tumor activity. Mechanisms of TEX-driven reprogramming might involve nucleic acids, especially double-stranded (ds)DNA. **Methods**: TEX isolated from supernatants of human tumor cells were identified as sEV, based on their size, endocytic origin and morphology. TEX treated with DNase/RNase cocktail were examined by transmission and cryo-electron microscopy and tested for biologic activity. DNA was extracted from enzyme-treated TEX, quantified by Qubit and analyzed for fragment sizes. The presence of genomic DNA in TEX was confirmed by PCR, and sequencing of the *TP53* gene fragment for a mutational signature was performed. **Results**: Enzymatic and microscopic studies of TEX showed that nucleic acids are present in the biocorona on the outer surface. Their removal interfered with the biocorona integrity. A short TEX exposure to DNase/RNase altered their morphology without impairing vesicle functions; longer treatments induced TEX re-organization into smaller membrane-bound vesicles. The TEX lumen contained long fragments of protected genomic DNA with a mutational signature reflecting that of the tumor. **Conclusions**: Nucleic acids present on the TEX surface support the vesicular integrity. The TEX lumen contains membrane-protected large (ds)DNA fragments with the mutational signature of the parent tumor. The presence of surface and luminal nucleic acids in TEX, and especially their mutational signature, suggests that TEX may serve as highly promising cancer-specific biomarkers.

## 1. Introduction

Tumor-derived extracellular vesicles are a subset of EVs produced by tumor cells. These EVs are present in all body fluids of cancer patients, representing a variably large fraction of all circulating EVs [1,2]. Tumors produce and release a variety of EVs differing in size that range from very small (<30 nm) to larger microvesicles (200–500 nm) and even larger apoptotic bodies (>1000 nm). These EVs differ in their biogenesis, as small EVs largely derive from late endosomes and multivesicular bodies (MVBs) in tumor cells, microvesicles are formed by “blebbing” of the cell membrane, and apoptotic bodies are products of dying tumor cells [3,4]. Once released by tumor cells into the intercellular space, EVs migrate to the vascular and lymphatic systems and circulate freely, reaching all tissues and all body fluids [5].

Among the various types of vesicles that tumors release, small tumor-derived EVs, which we call TEX, are of special interest in the emerging field of immuno-oncology. This is largely due to the perception that TEX play an important role as promising cancer biomarkers, but also because they serve as carriers and distributors of molecular/genetic information between tumor cells and all other cells in the tumor microenvironment (TME) [6]. The bioactive TEX cargo of proteins, lipids, glycans and nucleic acids approximates that of parent tumor cells [7]. TEX are morphologically indistinguishable from EVs produced and released by healthy cells, but their phenotypic, molecular, and genetic features are different [8]. Much of what is known about the origin, content and functions of TEX has been obtained from studies of cancer cell lines, which produce only tumor-derived EVs, allowing for the isolation of “pure” TEX from culture supernatants. The isolation of TEX from these supernatants can be accomplished by a variety of methods [9], and the detailed, reliable protocol for TEX isolation from cell supernatants and for their characterization has been published [10].

TEX, horizontally transfer and deliver a wide variety of biologically active molecules to the recipient non-malignant or malignant cells, altering their behavior [11,12]. This process, known as TEX-driven reprogramming, includes the transfer of proteins, lipids, glycans, and nucleic acids, resulting in major metabolic alterations of cells in the TME [11,12]. The presence of functional nucleic acids, mRNAs, micro RNAs, long non-coding RNAs and circular RNAs in EVs has long been accepted as evidence for EVs capability to shuttle genetic material between cells and serve as biomarkers of disease [13]. However, the presence in EVs of double-stranded genomic DNA is still being questioned today, despite accumulating evidence that confirms the 2014 report by Kahlert et al. that EVs from the plasma of patients with pancreatic cancer carried double-stranded (ds) genomic DNA with mutated *KRAS* and *p53* [14]. Since then, several studies have similarly reported that DNA in tumor cell-derived EVs represents the entire genome and reflects the mutational status of parental tumor cells [15,16,17]. As such, dsDNA in TEX assumes a key role as a biomarker in cancer detection as well as a major EV component responsible for horizontal information transfer and reprogramming of cells in the TME, driving them to metastatic phenotypes. Emerging evidence for the capability of TEX to reprogram different immune cells in the TME, converting them from anti-tumor to pro-tumor activity [18,19] emphasizes the great importance of TEX in immuno-oncology. There is a concern that the combination of immunological and genetic reprogramming mediated by TEX may promote metastasis and may be responsible for the resistance of cancers to immune therapies. Today, studies of the DNA content in TEX are of primary importance for: (i) providing convincing evidence of the dsDNA presence in TEX; (ii) discrimination between surface and luminal DNA in TEX; (iii) functional consequences that DNA in TEX imposes on immune and non-immune cells in the TME; (iv) evaluating the role of circulating tumor ctDNA vs. DNA in TEX as potential biomarkers of disease progression. Here, using a variety of DNA-specific assays and cryo-electron microscopy (cryo-EM), we assess the presence, content, distribution, and functional impact on recipient cells of DNA carried by TEX isolated from supernatants of two different human tumor cell lines. The experimental model we used is focused on molecular/genetic events driven by the DNA-carrying TEX that may ultimately lead to tumor progression and metastasis.

## 2. Materials and Methods

### 2.1. Cell Lines

Human head and neck squamous cell carcinoma cell lines UM-SCC47 (HPV(+) provided by Dr. Thoms Carey, University of Minnesota [20], PCI-13 (HPV(-) established at the University of Pittsburgh [20] and FaDu-Luc2 HPV(-) purchased from the American Type Culture Collection (Manassas, VA, USA) were cultured in Dulbecco’s Modified Eagle Medium (DMEM; Gibco, Thermo Fisher Scientific, MA, USA) supplemented with 10% (*v*/*v*) exosome-depleted, heat-inactivated fetal bovine serum (FBS; Gibco, Thermo Fisher Scientific), 100 U/mL penicillin, and 100 µg/mL streptomycin at 37 °C in a humidified incubator with 5% CO_2_. For production of tumor cell-derived exosomes (TEX), 2.5 × 10^6^ cells were seeded in 25 mL of medium in a T-175 flask and cultured for 72 h. The FaDu cell line was cultured in the same media and under the same conditions in the Pietrowska laboratory in Poland.

Jurkat T cells expressing surface CD8 were generated by Dr. H. Rabinowich (Department of Pathology, University of Pittsburgh, PA) and were cultured in RPMI-1640 medium (Thermo Fisher Scientific, MA, USA) supplemented with 10% (*v*/*v*) exosome-depleted, heat-inactivated fetal bovine serum (FBS, Gibco, Thermo Fisher Scientific), 100 U/mL penicillin, and 100 µg/mL streptomycin in a humidified incubator at 37 °C in 5% CO_2_. CD8^+^ Jurkat cells were periodically maintained in selection medium (RPMI with Geneticin). Prior to experimental use, CD8 expression was verified by flow cytometry. Routinely, 70–80% of cultured Jurkat cells were CD8^+^.

All cell cultures were regularly tested for Mycoplasma contamination using the Lonza Mycoplasma Detection Kit (cat. LT07-218, Lonza, Rockland, ME, USA) and found to be negative.

### 2.2. TEX Isolation and Characterization

TEX were isolated from culture supernatants or body fluids as previously described [21]. Briefly, pre-cleared supernatants were concentrated to 1 mL using Vivaspin centrifugal filters (100,000 MWCO; Sartorius, Bohemia, NY, USA) and applied to a Sepharose^TM^ CL-2B (cat. 17014005, Cytvia Sweeden AB, Uppsala, Sweeden) column for exclusion chromatography (SEC) performed as previously described [21,22]. The column was eluted with phosphate-buffered saline (PBS, Gibco, Thermo Fisher Scientific), and 1 mL fractions were collected. Fraction #4 containing the bulk of morphologically intact, non-aggregated vesicles [21,22] was concentrated using 100,000 NMWL Amicon^®^Ultra—0.5 mL centrifugal concentrators (cat. UFC510096, Merck Millipore Ltd., Darmstadt, GermanyProtein concentrations were determined using the BCA protein assay kit (Pierce Biotechnology, Rockford, IL, USA).

Transmission electron microscopy (TEM) was performed using SEC fraction #4 at the Center for Biologic Imaging, University of Pittsburgh, as previously described [22]. Vesicles were stained with 1% (*v*/*v*) uranyl acetate in ddH_2_O on copper grids and imaged using a JEOL JEM-1011 microscope. CryoEM was performed by Dr. James Conway at the Pittsburgh Center for Cryo-Electron Microscopy, University of Pittsburgh. Briefly, 3 µL of sample was pipetted onto freshly glow-discharged C-flat R2/1 Cu 300 mesh holey carbon grids (Protochips, Morrisville, NC, USA), blotted for 4 secs, and plunge-frozen into a 60:40 mixture of liquid propane and liquid ethane [23] using a Thermo Fisher Scientific (TFS: Waltham, MA, USA) Vitrobot Mark IV. Vitrified grids were stored in liquid nitrogen until imaging. Images were collected using low-dose methods on a TFS Titan Krios G3i operating at 300 kV with a Selectris energy filter and Falcon 4i camera under control of the EPU v3.7 software. The nominal magnification was 81,000× (pixel size of 1.51 Ångstroms at the sample), and images were collected in electron-counting mode with the energy filter slit width set to 10 eV and under focus ranging between −1.5 and −2.5 μm. Exposures used a total dose of ~30 electrons/Å2, and motion correction was performed with MotCorr2 [24] to remove stage drift.

Nanoparticle tracking analysis (NTA) was conducted to determine the concentration and size distribution of TEX using a NanoSight NS300 system (Malvern Instruments, Worcestershire, UK). Videos were captured and analyzed using NTA software (NanoSight NTA 3.4) with screen gain and detection thresholds set at 1 and 5, respectively. Each sample was measured five times, and the average particle size and concentration were calculated and reported. In addition, NTA was performed using ZetaView (Particle Metrix, Meerbusch, Germany) and the corresponding software (ZetaView 8.02.28) [25]. Polystyrene NanoStandards particles from Applied Microspheres GmbH with a known average size of 100 nm were used to calibrate the instrument prior to measurement. For each sample, 1 mL of the sample, diluted in 1 × PBS, was loaded into the cell, and the instrument measured each sample at 11 different positions throughout the cell. After automated analysis of all 11 positions and removal of any outlier positions, the mean, median, and concentration of the sample were calculated by the optimized instrument’s software. For each measurement, the instrument pre-acquisition parameters were set to a temperature of 23 °C, a sensitivity of 65, and a shutter speed of 100.

Western blotting was performed to analyze protein markers in TEX. Vesicle samples (5 µg protein) were lysed in Laemmli buffer (Bio-Rad Laboratories, Hercules, CA, USA), separated by 12% SDS-PAGE, and transferred onto PVDF membranes. Blots were probed with antibodies against CD9 (tetraspanin; cat. 13174; Cell Signaling, Danvers, MA, USA), endocytic protein markers ALIX (cat. MA5-32773; Invitrogen, Carlsbad, CA, USA) and TSG101 (cat. PA5-31260; Invitrogen, Carlsbad, CA, USA), and negative cytosol markers Grp94 (cat. 20292; Cell Signaling, Danvers, MA, USA) and Calnexin (cat. 2679; Cell Signaling, Danvers, MA, USA).

### 2.3. DNase/RNase Treatment

DNase I (cat. EN0521, ThermoFisher Scientific, Waltham, MA, USA), an endonuclease capable of degrading single- and double-stranded DNA, was combined with RNase A for the assessment of nucleic acid localization in the vesicle corona or lumen. Freshly isolated TEX (fraction #4; 1 × 10^10^ particles) were suspended in PBS with MgCl_2_ and incubated with a mixture of DNase I (0.05 U/μL) and RNase A (0.05 μg/μL; cat. EN0531 ThermoFisher, Waltham, MA, USA) for 5 or 10 min at room temperature (RT). The reaction was terminated by the addition of 50 mM EDTA (equal volume to MgCl_2_ buffer) and RiboLock RNase Inhibitor (1:1 with RNase A; cat. EO0381, ThermoFisher, Waltham, MA, USA). In preliminary experiments, TEX were co-incubated for 15 minutes with MgCL2 and EDTA buffer to show that the buffer had no effects on vesicle numbers or size.

### 2.4. DNA Isolation, Quantification, and Fragment Analysis

DNA was extracted from untreated and enzyme-treated TEX using the PureLink Genomic DNA Mini Kit (cat. K1820-00, ThermoFisher Scientific) according to the manufacturer’s protocols. The samples were eluted twice in 25 μL of elution buffer. Recovered DNA was normalized to the number of TEX recovered in fraction #4 and used for DNA quantification performed using the Qubit dsDNA High-Sensitivity Assay kit (cat. 32854, ThermoFisher Scientific). Fragment size analysis was conducted using the Agilent High Sensitivity DNA Bioanalyzer kit (cat. 5067-4626, Agilent, Santa Clara, CA, USA). The fragmentomics analysis was performed at the Hillman Cancer Center Genomics Facility, University of Pittsburgh.

### 2.5. PCR Confirmation of Genomic DNA Presence in TEX

Genomic DNA from FaDu cells was isolated using the Wizard^®^ SV Genomic DNA Purification System (cat. A2360, Promega, Fitchburg, WI, USA), according to the manufacturer’s protocol. Concentration and purity were assessed using a NanoDrop ND-1000 spectrophotometer (NanoDrop Technologies, Wilmington, DE, USA).

PCR amplification was performed using the OneTaq^®^ Quick-Load^®^ 2X Master Mix with Standard Buffer (Cat. M0486G, New England BioLabs, Ipswich, MA, USA) in a final reaction volume of 50 µL, containing 100 ng of genomic DNA as template. Amplification was carried out with the following primers, synthesized by Genomed S.A. (Warsaw, Poland):

- Forward primer: p53_for2: 5′-CTATGAGCCGCCTGAGGT-3′;
- Reverse primer: p53_rev2: 5′-GCTGTTCCGTCCCAGTAG-3′.

PCR reactions were conducted in a thermal cycler (model pegLab, VWR International, Radnor, PA, USA) under the following cycling conditions: initial denaturation at 95 °C for 5 min; followed by 30 cycles of denaturation at 95 °C for 30 s, annealing at 50 °C for 1 min, and extension at 68 °C for 1 min and 40 s. The final extension was performed at 68 °C for 5 min. PCR products were separated using 1% agarose gels in 0.5 × TBE buffer and visualized using ethidium bromide staining. The Quick-Load^®^ Purple 1 kb Plus DNA Ladder (Cat. N0550G, New England BioLabs, Ipswich, MA, USA) was used as a molecular size marker.

Amplified products were submitted for Sanger sequencing at Genomed S.A. (Warsaw, Poland). Sequencing was performed using the p53_rev2 primer, and results were provided electronically. Chromatograms were analyzed using Chromas LITE, 2.6.6 (Technelysium Pty Ltd., Brisbane, Australia).

### 2.6. EdU Labeling of EV-DNA

To label TEX-associated DNA, UM-SCC47 cells (2.5 × 10^6^) were incubated with 10µM EdU (5-ethynyl-2-deoxyuridine; cat. A10044, Invitrogen, Carlsbad, CA, USA) for 3 h. EdU-containing medium was then replaced with fresh medium, and cells were cultured for an additional 72 h to collect TEX containing EdU. Vesicles were fixed with 4% paraformaldehyde (PFA) and permeabilized with 0.01% Triton X-100 for 15 min at RT and then were labeled using the Click-iT™ Plus Edu Alexa Fluor™ 647 Imaging Kit (cat. C10640, Invitrogen, Carlsbad, CA, USA) following the manufacturer’s instructions for single TEX-DNA imaging.

### 2.7. Functional Evaluation of DNase/RNase-Treated TEX

To evaluate potential functional changes in TEX treated with the DNAse/RNase cocktail, uptake of treated vs. untreated TEX by CD8+ Jurkat T cells and apoptosis of CD8^+^ Jurkat T cells were evaluated.

To evaluate TEX uptake by Jurkat T cells, TEX were labeled with the membrane dye MemGlow™ 590 (cat. MG03-02, Cytoskeleton, Inc., Denver, CO, USA) according to the manufacturer’s instructions. Briefly, TEX (30 µg) in 100 µL PBS were mixed with a 100 nM solution of MemGlow™ 590, working quickly (<20 s) to prevent aggregation, and incubated for 10 min at RT. Labeled TEX were washed ×5 with PBS using 100,000 NMWL Amicon^®^Ultra—0.5 mL—filters and resuspended in 100 µL PBS. To monitor TEX uptake by Jurkat T cells, 10 µL aliquots of freshly labeled untreated or DNase/RNase -treated TEX were co-incubated with 1 × 10^5^ CD8^+^ Jurkat cells in 100 µL RPMI for 0, 15, 30, or 60 min at 37 °C. Negative controls included Jurkat cells treated with the dye in PBS only. Cells were washed x3 with PBS, and the TEX uptake was quantified using flow cytometry (CytoFLEX, Beckman Coulter, Brea, CA, USA).

To measure TEX-induced apoptosis, CD8 + Jurkat cells (1 × 10^5^/well) were plated in U-bottom 96-well plates in RPMI medium. Cells were incubated for 6 h with untreated or DNase cocktail-treated (for 5 or 10 min) TEX (2.5, 5.0, or 10 µg protein). Controls included Jurkat cells in PBS or Jurkat + anti-FasL Ab. Apoptosis was assessed using the Alexa Fluor 488 Annexin V Apoptosis Detection Kit (cat. V13245, InvitrogenCarlsbad, CA, USA) in a CytoFLEX flow cytometer (Beckman Coulter, Brea, CA, USA).

### 2.8. Confocal Microscopy of Labeled sEV

Jurkat cells incubated with EdU and/or PKH26-labeled TEX (untreated or DNase cocktail-treated) were harvested, washed, and attached to slides using cytospin. Cells were fixed with 4% PFA (15 min, RT), stained with SPY555-actin dye (cat. SC202, Spirochrome, Stein am Rhein, Switzerland, 1:1000 dilution) for 1 h at RT, washed, and counterstained with DAPI. Images were acquired on a Nikon confocal microscope (Melville, NY, USA) using a 60x objective.

### 2.9. Zeta Potential Measurements

To measure the zeta potential of TEX samples, an IZON nanoparticle characterization system equipped with an NP150 nanopore was used. TEX suspensions were divided into two groups: one treated with DNase cocktail for 5 or 10 min, and the other left untreated in PBS as a control. Following treatment, samples were diluted in the measurement electrolyte to achieve optimal concentration for analysis. Diluted samples were equilibrated at RT for 10 min, and then the Zeta potential was measured at 25 °C. The IZON instrument utilizes tunable resistive pulse sensing (TRPS) through the NP150 nanopore to determine particle electrophoretic mobility. The Zeta potential was calculated from electrophoretic mobility using the Smoluchowski equation. Changes in surface charge were compared between treated and untreated TEX samples.

### 2.10. Statistical Analysis

The experimental design used for 3 independent experiments with DNase/RNase-treated TEX is illustrated in Figure S1. The study included 3 experimental groups: 0 no treatment (PBS control), 5 min treatment and 10 min treatment. Paired two-sample T-tests were used to separately assess the difference between each treated group vs. the control. Statistical analyses were performed using GraphPad Prism 10.0. All data sets were represented as means ± SD (shown with error bars). *p*-values ≤ 0.05 were considered significant.

## 3. Results

### 3.1. The Characteristics of Small Extracellular Vesicles Isolated from Supernatants of Tumor Cell Lines

**Figure 1:**
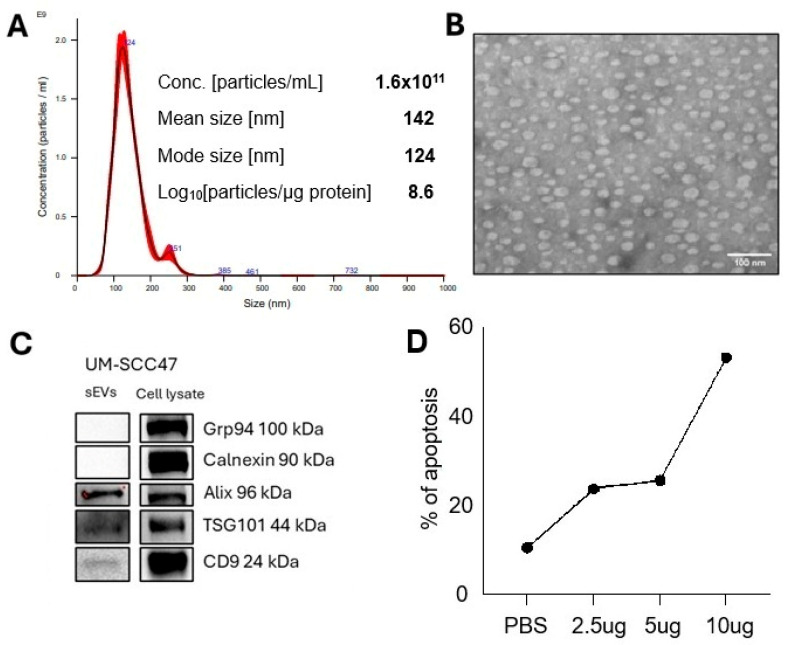
**Characteristics of isolated TEX.** (**A**) size, concentration and the log ratio particles/µg protein (NTA), (**B**) vesicle morphology (TEM), (**C**) the presence of ALIX and TSG101 suggests endocytic origin of the vesicles (WBs of vesicles and cell lysate), (**D**) apoptosis of Jurkat T cells (1 × 10^5^) co-incubated with TEX at increasing protein concentrations for 6 h.

These vesicles were isolated by size exclusion chromatography (SEC) as previously described [21] from supernatants of tumor cell lines and were evaluated for size (NTA, TRPS), morphology (TEM), the potential endocytic origin (positive for ALIX, TSG101 in the absence of cytosolic proteins, e.g., calnexin, grp94), and biologic activity (induction of apoptosis in activated T cells), as illustrated in Figure 1. Based on these characteristics, the vesicles isolated from tumor cells were classified as small extracellular vesicles sEV [26], which we call TEX.

### 3.2. TEX Carry DNA on the Vesicle Surface

**Figure 2:**
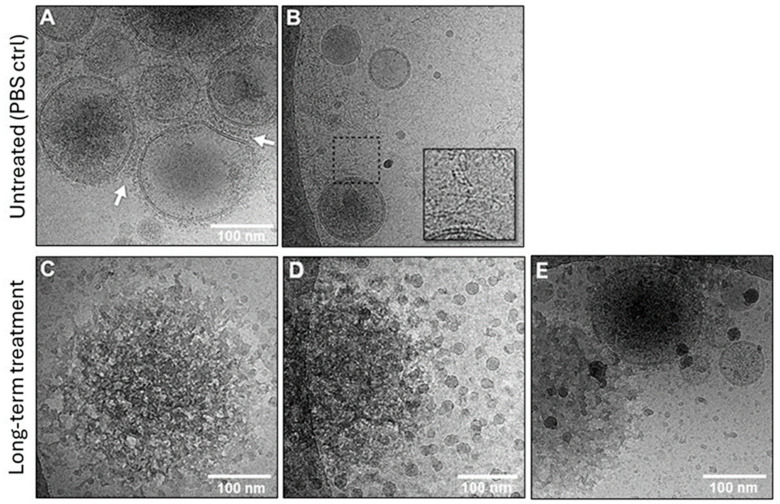
**DNA on the vesicle surface and TEX morphology.** In (**A**,**B**), cryo-EM images of TEX before treatment with the DNase/RNase cocktail. The arrows in (**A**) indicate the protein “biocorona” formed around the vesicular double membrane. In (**B**), the outlined area indicates the presence of long strands of the material loosely associated with vesicles. In (**C**–**E**), TEX after long-term (≥15 min) treatment with the DNase/RNase cocktail: in (**C**), a single vesicle with a disintegrating outer surface; in (**D**), the surface of the vesicle treated with the enzymatic cocktail is dissociating into numerous smaller vesicles. In (**E**), some TEX stripped of the surface biocorona retain their characteristic double-membrane morphology.

Vesicles isolated from supernatants of tumor cell lines were evaluated for the presence of nucleic acids on the vesicle surface and in the vesicle lumen. TEX were incubated in the presence of DNase/RNase cocktail for various time periods (5, 10 and >15 min) or with PBS as a control. Using cryo-EM to view PBS-treated and enzyme-treated TEX, we observed that the DNase/RNase treatment for 15 min or longer resulted in “stripping” from the surface of larger vesicles of material, which dissociated into masses of smaller vesicles. Figure 2A,B show that prior to DNase/RNase treatment, TEX are enclosed by a double membrane with the corona of closely packed surface proteins. Strands of material that may be nucleic acids are loosely associated with the vesicles in the cryoEM images of these vesicles (Figure 2B). In TEX treated with DNase/RNase cocktail for ≥15 min, the surface biocorona is disintegrating, releasing masses of small vesicles from the TEX surface (Figure 2C,D). Importantly, as illustrated in Figure 2E, the double membrane surrounding TEX treated with the enzyme cocktail for >15 min remained intact, preventing damage to the luminal content of the vesicles. These observations led to the conclusion that prolonged exposure of TEX to RNase/DNase cocktail results in the disintegration of the surface biocorona but leaves the membrane-bound TEX largely intact (Figure 2E).

### 3.3. DNase Treatment Alters TEX Morphology

**Figure 3:**
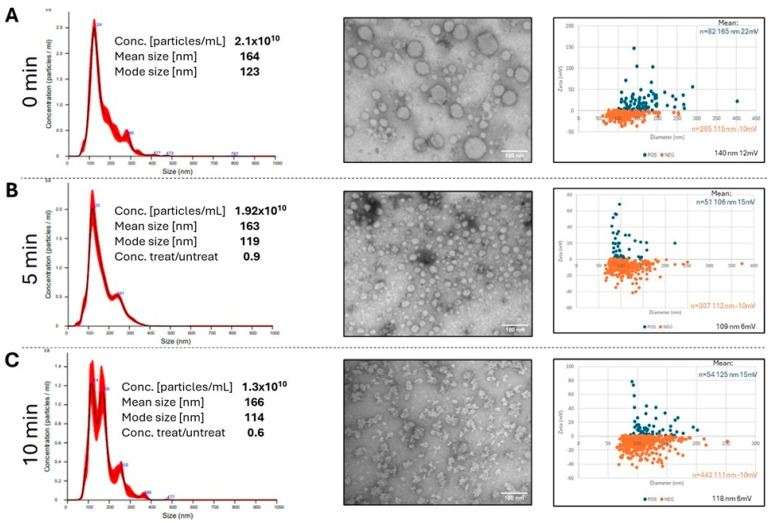
**Changes in TEX numbers and size before tratement** (**A**), **and following 5 min** (**B**) **or 10 min** (**C**) **treatment with the DNase/RNase cocktail relative to PBS control.** The NTA plots (**left panel**) are representative of 3 independent experiments as described in Figure S1. The decreasing ratios of treated/untreated TEX numbers suggest a loss of TEX during enzymatic treatments. The representative TEM images (**center panel**) of negatively stained TEX illustrate changes in vesicle size and shape after 0, 5 or 10 min of enzymatic treatments. Time-dependent changes in the TEX zeta potential and size are shown in the (**right panel**).

Our initial results suggested that the DNase/RNase treatment of TEX for more than 15 min caused disruption of larger vesicles, leaving behind membrane-bound smaller vesicles. To refine this approach, TEX were subjected to DNase/RNase digestion for 0 (PBS control), 5 min and 10 min. While the mean particle size measured by NTA remained unchanged (~120 nm) during the short enzymatic treatment (Figure 3, left panel), changes in the NTA profile toward smaller particles are evident after treatment. Also, the ratio of treated/control vesicle numbers decreased to 0.9 at 5 min and to 0.6 at 10 min, suggesting a loss of some vesicles during the treatment (Figure 3, left panel). TEM images of negatively stained vesicles (Figure 3, center panel) revealed subtle time-dependent changes in the vesicular appearance of TEX treated with DNase/RNase, which became noticeable after 10 min exposure. Additionally, the NTA size distribution profiles (Figure 3, left panel), along with the zeta potential measurements (Figure 3, right panel), indicated that DNA removal led to changes in the TEX size and a shift in the surface charge from positive to negative. These changes may be consistent with increased vesicle stickiness upon a loss of nucleic acids.

**Figure 4:**
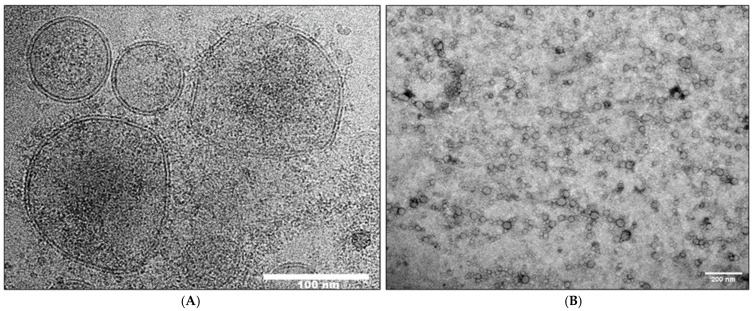
**Representative cryo-EM and TEM images of TEX after 10 min treatment with the DNase/RNase cocktail.** In (**A**), cryo-EM shows alterations in the vesicle shape and the release or “stripping” of materials from the vesicular surface. The integrity of the vesicle double membrane appears to be intact. In (**B**), TEM of negatively stained TEX suggests formation of chain-like arrangements of vesicles following 10 min treatment with the enzyme cocktail.

The cryo-EM images of enzymatically treated TEX confirmed that time-dependent progressive morphological changes were occurring on the TEX surface membranes. After 5 min treatment, strands of the material that appeared to be nucleic acids outside of the vesicle surface (see Figure 2B) were mostly, although not completely, gone, confirming their identity as nucleic acids. Treatments of TEX with the enzyme cocktail for 10 min led to changes in the vesicle shape and size, but without any obvious evidence for breaks in the vesicular double membrane. Figure 4A illustrates distortions in the shape of larger vesicles and “stripping” of the material associated with the vesicle surface. In addition, TEM images of negatively stained TEX after 10 min treatment with the enzyme cocktail (Figure 4B) indicate the formation of chain-like vesicle clusters, possibly because of changes in the vesicle surface that promote vesicle aggregation.

Using on-bead flow cytometry of TEX co-incubated with the enzyme cocktail for 10 min, phenotypic changes in the TEX surface proteins were observed. Specifically, expression levels of CD63, CD81, PD-L1 and TGF-β slightly increased (not significant) on the surface of DNase/RNase-treated TEX relative to untreated controls. These preliminary flow cytometry-based experiments further suggest that even the short (10 min) enzymatic treatment alters the vesicle surface.

**Figure 5:**
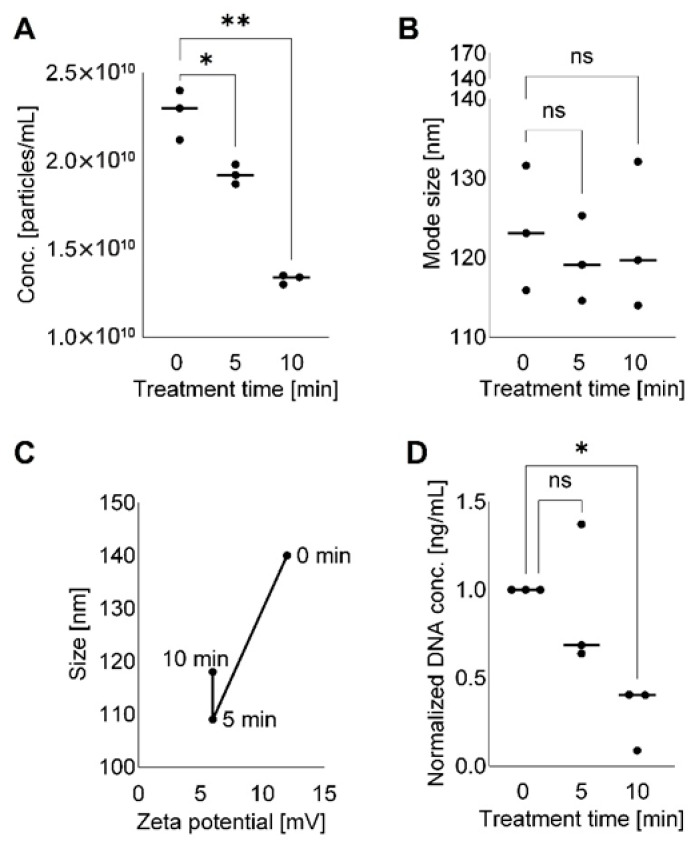
**Analysis of time-dependent changes in the number, size and DNA content of TEX incubated with DNase/RNase cocktail for 5 min and 10 min relative to untreated control (0 min).** In (**A**), decreasing numbers of TEX after short-term treatments: * *p* = 0.05; ** *p* = 0.009. In (**B**), changes in TEX size after treatment with the DNase/RNase cocktail: ns = no significant difference. In (**C**), changes in the size and Zeta potential of enzyme-treated TEX. In (**D**), Changes in DNA concentrations of TEX before and after treatment with enzymes. Data shown in (**A**,**B**,**D**) are from three independent experiments as indicated in Figure S1 and are normalized to 1 ml of the SEC fraction #4 in (**A**,**B**) and to time 0 in (**D**).

In aggregate, microscopic and phenotypic examination of DNase/RNase-treated TEX suggested that nucleic acids were present on the TEX surface, likely in association with the lipo-proteins that form the vesicle “corona” [27]. Further, the enzymatic removal of nucleic acids from the vesicle surface resulted in the loss of vesicular morphology, which progressed from mild to extensive upon prolonged exposure to the enzymes. Figure 5 presents the analysis of time-dependent enzymatically induced changes in TEX numbers, size, shape, Zeta potential and DNA content based on the results of 3 independently performed experiments as illustrated in Figure S1. The analysis was designed to address two hypothetical pairwise comparisons: (i) untreated control vs. 5 min treated TEX and (ii) untreated control vs. 10 min treated TEX. The results indicate that significant shits in vesicle numbers, but not the vesicle size, occurred during the 5 min as well as 10 min treatment with DNase/RNase cocktail. Changes in vesicle DNA content were significant after 10 min treatment with DNase/RNase.

### 3.4. The Presence of Luminal DNA in TEX

**Figure 6:**
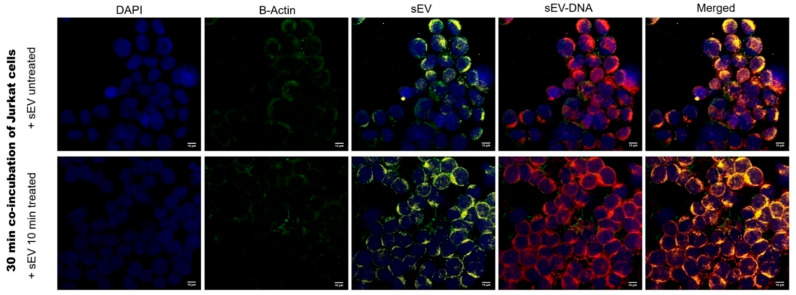
**Confocal microscopy of Jurkat T cells co-incubated for 30 min with TEX carrying EdU-DNA.** The EdU-DNA labeled TEX were either treated or not treated with the DNase/RNase cocktail for 10 min. TEX entering recipient Jurkat cells are labeled yellow, EdU-labeled DNA in TEX is labeled red and nuclei are blue. The background staining was subtracted from that of PBS (no TEX) control. Mag = 60×. Each bar = 10 µm.

To show that DNA is carried by TEX, EdU (5-ethynyl-2’-deoxyuridine) was used to label DNA in tumor cells. Next, EdU-DNA-containing TEX produced by these tumor cells were isolated from supernatants, permeabilized and stained with the PKH-26 dye to label vesicular membranes. Confocal microscopy of Jurkat T cells co-incubated with EdU-DNA TEX, which were untreated (PBS control) or treated with the enzyme cocktail for 10 min, indicated that sEV carrying EdU-labeled DNA were internalized by recipient Jurkat T cells within 30 min (Figure 6). The 10 min enzymatic treatment expected to remove surface-associated DNA did not visibly affect the intensity of EdU-DNA staining in the vesicles or the entry of labeled TEX into Jurkat cells. We concluded that EdU-DNA was largely located in the sEV lumen and was protected from enzymatic digestion by the surface membrane (Figure 6). Importantly, the 10 min enzymatic treatment did not interfere with the sEV signaling or entry of sEV into Jurkat T cells.

### 3.5. Treatment of TEX with DNase/RNase Does Not Impair Their Biologic Activity

Because of the observed changes in the morphology of TEX after 10 min of enzymatic treatment, we considered that the functionality of the vesicles may also be altered. Hence, we evaluated the uptake of TEX by Jurkat T cells and the TEX capability to induce apoptosis of Jurkat T cells. As shown in Figure S2A, when Jurkat T cells were co-incubated with TEX treated with DNase/RNase cocktail for 5 or 10 min, their entry was initially slower compared to untreated TEX, but by 30 min, all cells were positive. The mean fluorescence intensity of cells co-incubated with enzyme-treated TEX was somewhat lower than that seen in controls, suggesting a lower content of stained membranous materials in the treated TEX. As also previously reported [28], TEX were internalized by T cells within 5–30 min, and their uptake was only briefly delayed by the 10 min treatment with DNase/RNase.

Figure S2B shows that after 10 min enzymatic treatment, TEX were able to induce apoptosis in Jurkat cells and showed a mildly increased potential to trigger necrosis compared to untreated TEX. The results of apoptosis assays are especially important given our previously reported evidence that elevated levels of T cell apoptosis mediated by tumor-derived EVs in cancer patients are associated with disease activity [29]. Here, even after the removal of DNA from the vesicle surface, TEX were able to induce metabolic changes in recipient Jurkat cells, resulting in apoptosis of activated immune effector cells, as expected [30]. Thus, despite changes in their vesicular morphology after 10 min DNase/RNase treatment, TEX retained their biological activity.

### 3.6. TEX Carry Long DNA Fragments in the Lumen

**Figure 7:**
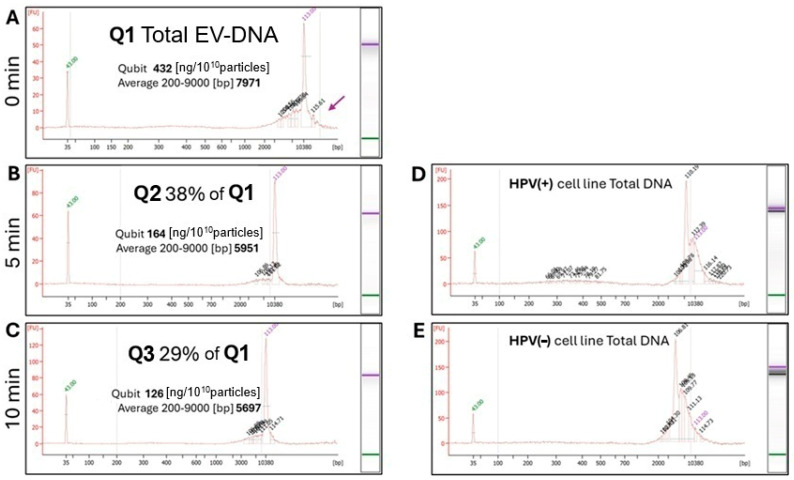
**Quantitative (Qubit) and qualitative (Bioanalyzer) results of DNA levels in the untreated TEX and TEX treated with DNase/RNase for 5 or 10 min.** In (**A**), Q1 = Total DNA normalized to vesicle number in untreated TEX obtained from UM-SCC47 cells; In (**B**), Q2 = 38% of total DNA remaining in TEX after 5 min treatment with the enzyme cocktail. In (**C**), Q3 = 29% of total DNA remaining in TEX after 10 min treatment. In (**D**,**E**), cellular DNA from HPV(+) UM-SCC47 cell line and HPV(−) PCI-13 cell line, respectively, illustrate results of the fragmentomic analysis of genomic DNA. Note the presence of large DNA fragments (>11,614 bp and 11,473 bp, respectively).

To show that TEX carry luminal DNA in addition to surface-associated nucleic acids, DNA was extracted from untreated and enzyme-treated TEX and total DNA was quantified by Qubit (Figure 7). This analysis showed that in TEX treated with DNase/RNase for 5 min 38% of total vesicular DNA was protected and preserved in the TEX relative to the control (Q1 in Figure 7B). After 10 min treatment with the enzyme cocktail 29% of Q1 total DNA remained associated with the vesicles (Figure 7C), representing DNA protected from enzymatic digestion. The data suggest that considerable amounts of vesicle-associated DNA are lost upon longer exposure of vesicles to the enzyme cocktail. The Bioanalyzer results showed that untreated TEX contained an excess of long DNA fragments (>11,561 bp) in addition to shorter fragments with an average length of 7971 bp. In comparison to genomic DNA from UM-SCC47 cells, luminal DNA in TEX obtained from the same cells was similarly fragmented, with perhaps somewhat shorter fragments (compare Figure 7A to Figure 7D). In TEX treated for 10 min, there were fewer long DNA fragments relative to untreated sEV. The fragmentomic analysis of DNA in the TEX lumen showed that, like genomic DNA in parental cells, it predominantly contains long DNA fragments ranging in length from 3000 to 10,000 base pairs (bp).

### 3.7. DNA in the TEX Lumen Reflects Mutations Present in Parental Cell DNA

**Figure 8:**
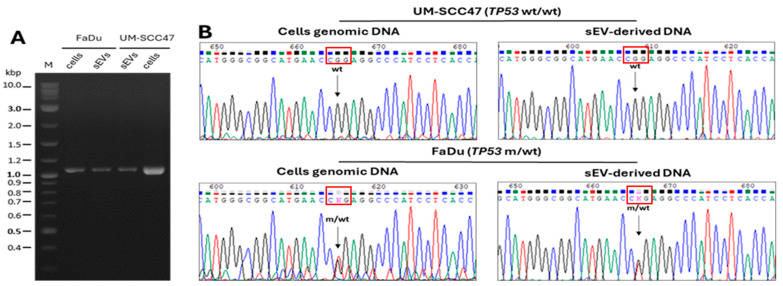
**Representative chromatogram fragments of *TP53* gene sequencing from UM-SCC47 and FaDu cell lines.** In (**A**), PCR amplification of the *TP53* gene fragment using genomic DNA from FaDu and UM-SCC47 cells and DNA isolated from their respective TEX. The expected size of the PCR product is 1062 bp. PCR products were separated on a 1% agarose gel and visualized with ethidium bromide. The Quick-Load^®^ Purple 1 kb Plus DNA Ladder (M) was used as a molecular size marker. In (**B**), Fragments of chromatograms from sequencing of PCR products of the *TP53* gene obtained from genomic DNA and TEX-derived DNA of UM-SCC47 and FaDu cells. Sequencing results were provided in electronic format and visualized using Chromas LITE, version 2.6.6. Mutation CGG to CTG (Arg to Leu) in the FaDu cell line and in TEX produced by FaDu cells is indicated in red box.

Of the two head and neck cancer cell lines cultured, UM-SCC47 and FaDu, the latter is known to harbor a *p53* gene mutation. The cell supernatants were used for TEX isolation. Genomic DNA from UM-SCC47 and FaDu cell lines was isolated, and following PCR amplification of the *TP53* gene fragment, the products were submitted for sequencing. The nucleotide sequence of amplified PCR products is shown in Figure 8. We searched for *TP53* gene mutations within TEX-derived DNA and compared them to those found in parental cell genomic DNA. Mutant *TP53* sequences were identified in both parental and TEX DNA (Figure 8), confirming that vesicular DNA carries the mutational signature seen in the parent cell genome. Thus, tumor-cell-derived TEX carry and transport genomic DNA with a mutational signature of the tumor.

## 4. Discussion

In agreement with several reports in the literature, this study reports on the presence of tumor cell-derived dsDNA in the lumen of TEX produced by human tumor cell lines with the identified mutations [15,17,31]. In addition, we provide evidence for the presence on the vesicle surface of DNA released by tumor cells into supernatants and incorporated into the external vesicular “biocorona” composed of a mix of extravesicular components termed “EV-interactome” [27]. It has been previously reported that the biocorona of EVs contained nucleic acids, including protein-associated DNA [32]. In our hands, a brief enzymatic digestion of TEX with the DNase/RNAase cocktail suggested that extravesicular DNA may be involved in maintaining vesicular integrity when complexed with RNA and proteins, forming the nucleoprotein biocorona surrounding the vesicles. Interestingly, cryo-EM images of TEX treated for extended periods of time with the DNase/RNase cocktail indicated their propensity to dissociate into smaller vesicular components, which may be exomeres [33]. This suggests that the surface-associated nucleic acids, forming a nucleoprotein corona together with the double lipoprotein membrane, play a role in sustaining vesicular morphology. It is also possible that the vesicle disintegration into smaller particles that is universally seen in preparations of sEV isolated from human plasma by TEM represents a mechanism that facilitates the uptake of vesicles by cells of the reticuloendothelial system and promotes their removal. The importance of the extra-vesicular biocorona for the preservation of vesicle morphology is important, because it suggests that inter-cellular interactions mediated by TEX depend on their integrity, size, and surface constituents.

The presence in the TEX lumen of genomic dsDNA with the same mutational signature that is seen in the parent tumor strongly supports the role of TEX as biomarkers of the tumor presence and cancer progression. The fact that intraluminal dsDNA is not fragmented and consists of long DNA fragments ranging in length from 3000 to 10,000 bp is especially significant. It means that DNA in the TEX lumen is protected from enzymatic cleavage in plasma and can be more readily and reliably sequenced than circulating tumor ctDNA. This, in turn, suggests that TEX isolated from body fluids of patients with cancer are likely to be a preferred source of cancer biomarkers relative to circulating soluble proteins or nucleic acids, which undergo proteolytic modifications and enzymatic cleavage. The available data indicate that the numbers of TEX are increased in plasma of patients with cancer [29] and that TEX can be isolated from cancer plasma in numbers adequate for DNA sequencing (e.g., 1 × 10^10^ to 1 × 10^11^ particles per 1 mL of plasma) and for the detection of mutated alleles. The potential advantage of using TEX in place of ctDNA for the detection of cancer lies in the availability of intact genomic DNA in the lumen vesicles, which is non-fragmented and which faithfully reproduces the mutational status of the tumor. We and others estimate that about 50% of vesicle -associated DNA is in the “biocorona” on the vesicle surface [32]. Since much of this DNA originates in the EV-interactome, it probably contains mostly circulating free DNA (cfDNA), although the circulating tumor DNA (ctDNA) may also be a component of the biocorona. As such, it enhances the value of TEX as a source of tumor-derived DNA, which, by its association with proteins in the biocorona, might also be protected from degradation.

Roughly half of vesicle-associated DNA is found in the TEX lumen. While in supernatants of cultured tumor cells, all vesicles are tumor-derived, TEX in body fluids of cancer patients represent a broadly variable percentage of total circulating EVs [34]. TEX isolation and their separation from non-malignant EVs have previously been successfully accomplished using antigen-specific immune capture [35], but this is a complex and not readily implemented procedure. With the emerging evidence of the TEX capability to deliver genetic material and potentially reprogram non-malignant cells, TEX isolation from the body fluids of cancer patients may not be necessary [36]. It is likely that a proportion of total plasma EVs that carry mutated DNA of the tumor could serve as cancer-specific biomarkers without resorting to “tumor-informed” analysis of ctDNA.

Considering their diverse roles and robust nature, TEX seem to be an ideal biomarker resource, and, as compared to circulating tumor cells (CTCs) and ctDNA, they offer unique advantages as liquid tumor biopsy: (i) TEX are proactively and selectively secreted by cells rather than being passively released as a consequence of necrosis or apoptosis, and tumors usually secrete an excess of vesicles [37]; (ii) TEX and their contents are comparatively stable because of their protective lipid-bilayer structure [38,39]; (iii) TEX are nano-sized and circulate freely crossing all organ barriers [40], which makes them ubiquitous in all body fluids, and can be efficiently enriched by many methods [41]; (iv) sEV comprise a diverse molecular cargo containing information about the cell of origin, which can be useful for the discovery of cancer-specific biomarkers [42]; (v) the surface and luminal DNA contents of TEX in cancer plasma enhances their value as cancer-specific biomarkers, since the accessibility of genomic mutated DNA in vesicles facilitates liquid biopsy analysis and eliminates its dependence on “tumor informed” ctDNA approach.

In the context of the ongoing discussion about the advantages and disadvantages of CTC, ctDNA and TEX as cancer-specific biomarkers, our data provide insights into the content, localization, and role of cancer cell-derived DNA associated with and carried by TEX. The DNA content of TEX lumen, whether mutated or wild type, and the DNA presence on the TEX surface, potentially stabilizing vesicular-cellular interactions critical for TEX functional activity, positions TEX as highly promising cancer biomarkers of the future.

## Data Availability

The dataset(s) supporting the conclusions of this article is (are) available upon request to the corresponding author.

## Supplementary Materials

The following supporting information can be downloaded at https://www.mdpi.com/article/10.3390/cells15060512/s1. Figure S1. Schematic representation of experimental design; Figure S2. Functional assays of DNase/RNase-treated TEX.
