# Supplementary material for: Nucleic Acids on the Surface and Lumen of Tumor-Derived Small Extracellular Vesicles as Potential Cancer Biomarkers
Source: Cells. 2026 Mar 13;15(6):512. doi: 10.3390/cells15060512 (PMC13025544; doi:10.3390/cells15060512)
Supplement: Supplementary file 1 [file cells-15-00512-s001.zip › cells-4175559-supplementary.pdf]

Figure S1. Schematic representation of experimental design. Tumor-derived extracellular vesicles (TEX) were isolated from the supernatant of the same cell line and subjected to DNase/RNase treatment for 0 min (PBS control), 5 min, or 10 min. For each independent experiment (labeled 1,2,3), TEX were isolated by SEC and were divided into three aliquots corresponding to 0, 5, and 10 min treatment conditions. The entire experiment was repeated three times on different days (n = 3; biological replicates).

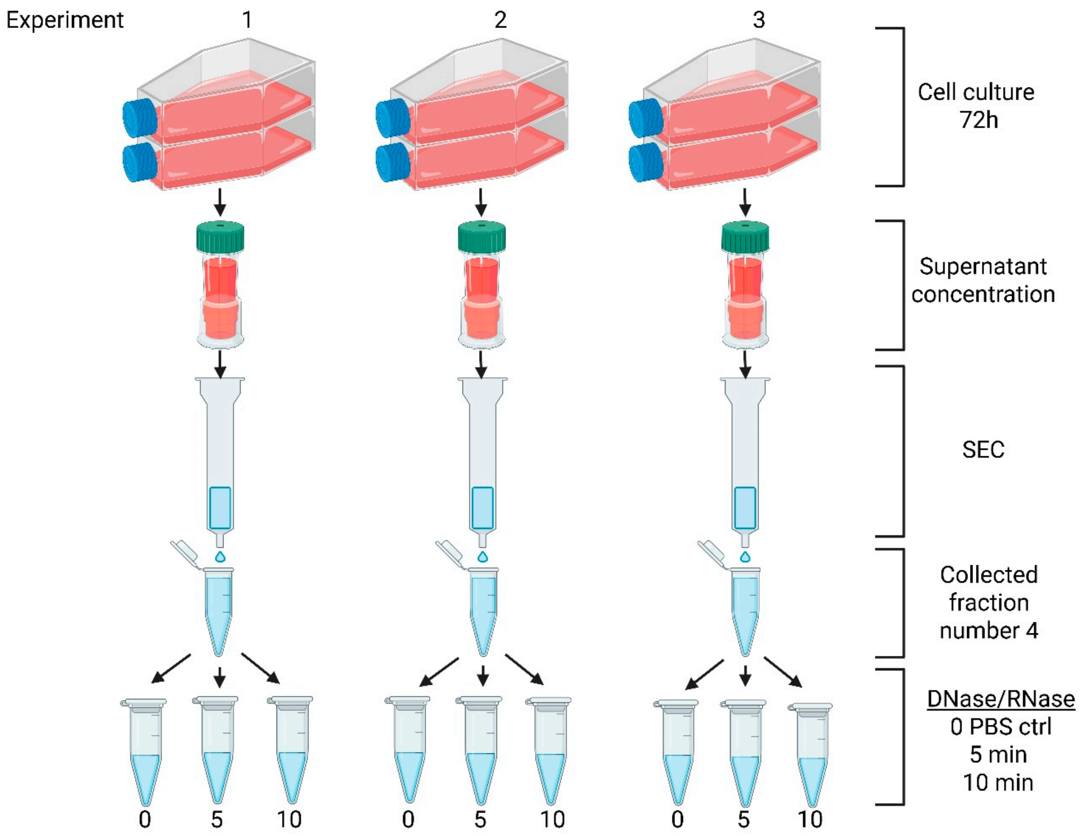

Figure S2. Functional assays of DNase/RNase-treated TEX. In **A,** TEX uptake by CD8$^+$ Jurkat T cells. Untreated or DNase/RNase-treated TEX were labeled with MemGlow™ 590 and co-incubated with CD8$^+$ Jurkat T cells for 0, 15, 30, or 60 min at 37°C. TEX uptake was quantified by flow cytometry and the results are presented as the percentage of MemGlow™-positive cells (*left panel*) and mean fluorescence intensity (MFI; *right panel*). Negative controls included Jurkat cells exposed to PBS with dye alone. In **B,** TEX-induced apoptosis. CD8$^+$ Jurkat T cells were incubated for 6h with untreated or DNase/RNase-treated TEX (5- or 10-min treatment) at increasing protein concentrations (2.5, 5.0, or 10 µg). Apoptosis was assessed by Annexin V staining and flow cytometry. Control conditions included Jurkat cells treated with PBS (negative control) or anti-FasL antibodies (positive control).

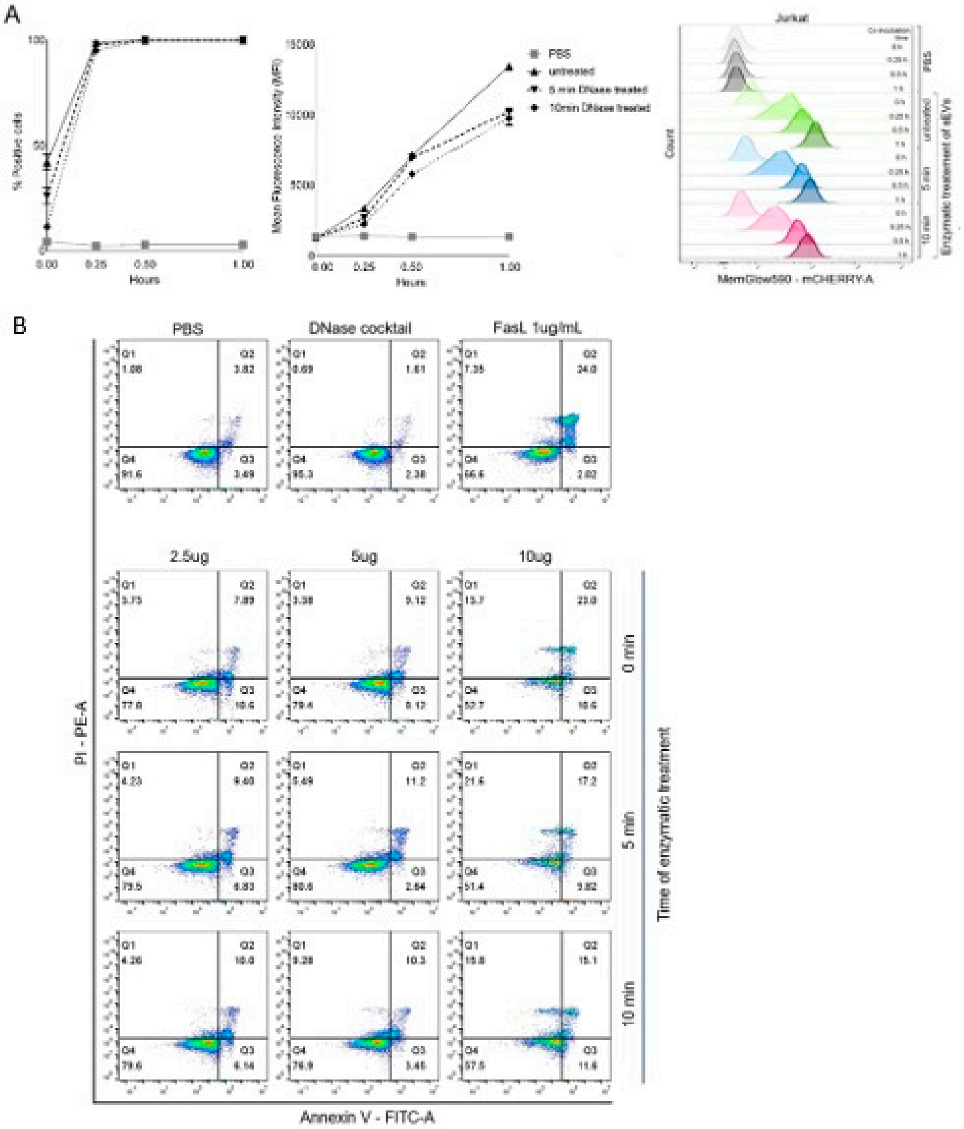

Western Blots RAW for TEX associated markers and for cell lysate

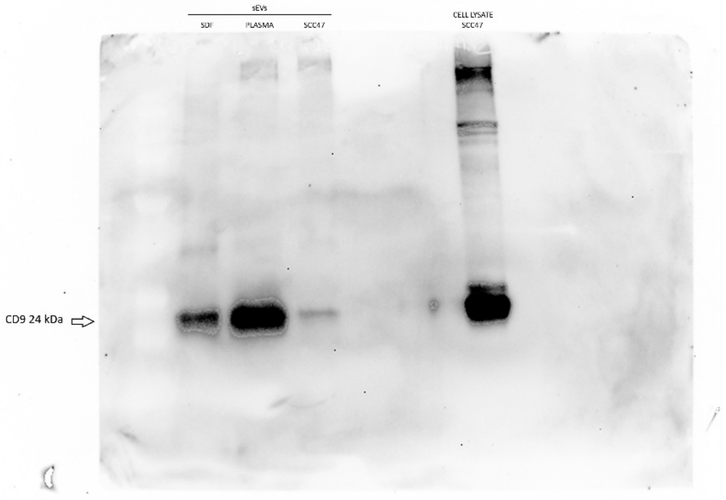

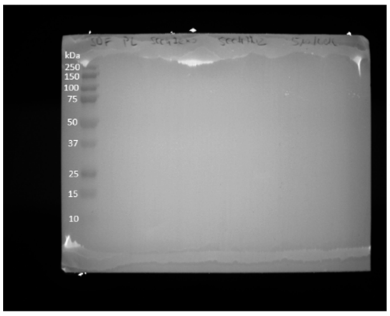

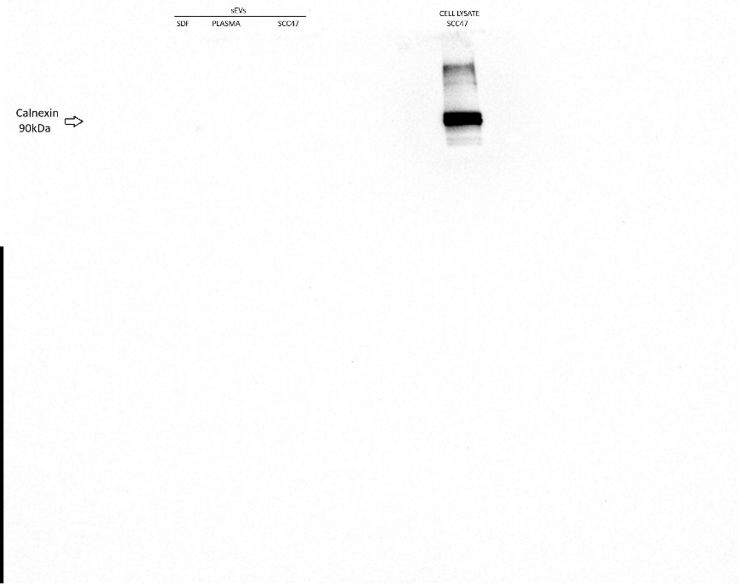

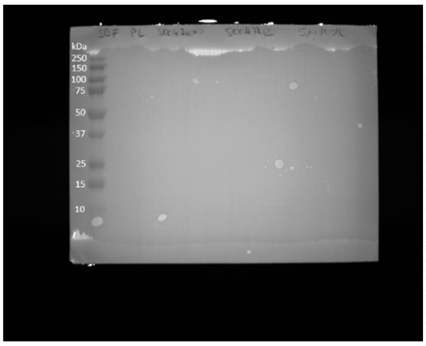

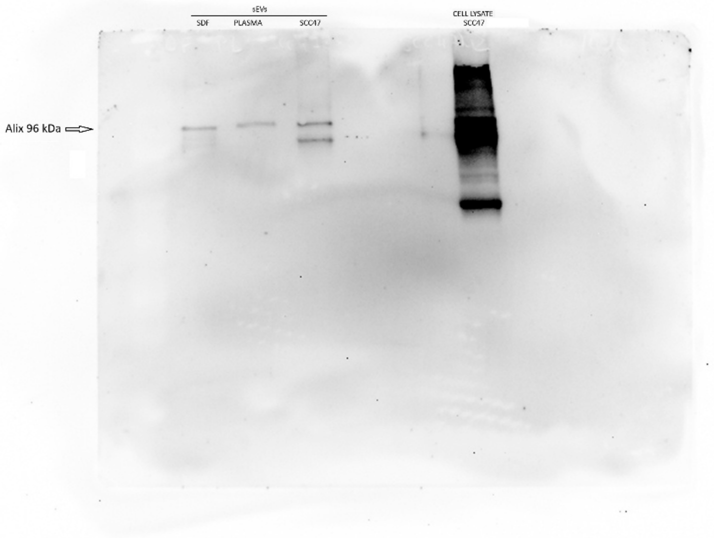
sEVs
SDF
PLASMA
SCC47
CELL LYSATE
SCC47
Alix 96 kDa

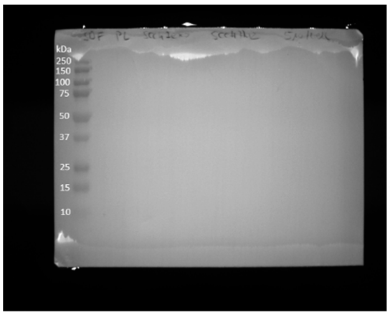
kDa
250
150
100
75
50
37
25
15
10

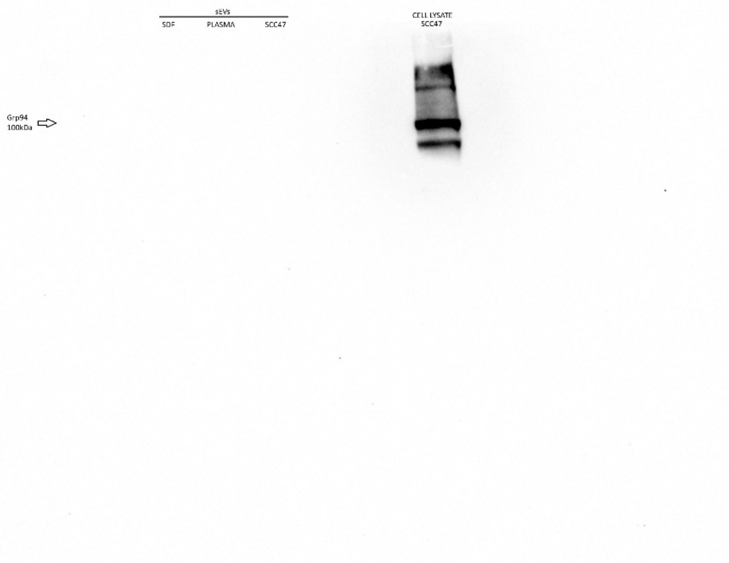
sEVs
SDF
PLASMA
SCC47
CELL LYSATE
SCC47
Grp94
100kDa

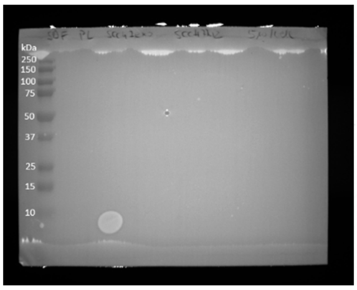
kDa
250
150
100
75
50
37
25
15
10

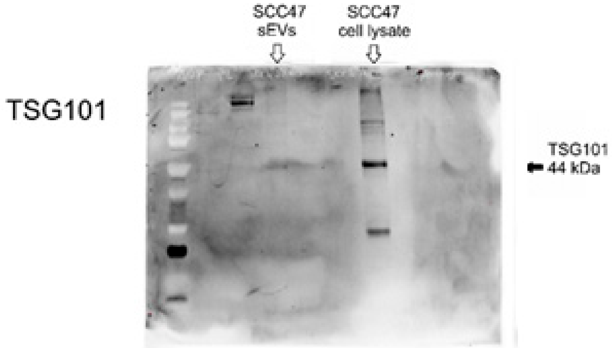
TSG101
SCC47
sEVs
SCC47
cell lysate
TSG101
44 kDa
